# Expression pattern and diagnostic value of ferroptosis-related genes in acute myocardial infarction

**DOI:** 10.3389/fcvm.2022.993592

**Published:** 2022-11-03

**Authors:** Jiahe Wu, Huanhuan Cai, Zhe Lei, Chenze Li, Yushuang Hu, Tong Zhang, Haoyan Zhu, Yi Lu, Jianlei Cao, Xiaorong Hu

**Affiliations:** ^1^Department of Cardiology, Zhongnan Hospital of Wuhan University, Wuhan, China; ^2^Institute of Myocardial Injury and Repair, Wuhan University, Wuhan, China

**Keywords:** acute myocardial infarction, ferroptosis, bioinformatics analysis, biomarker, ROC analysis

## Abstract

**Background:**

Ferroptosis is a form of regulatory cell death (RCD) caused by iron-dependent lipid peroxidation. The role of ferroptosis in the process of acute myocardial infarction (AMI) is still unclear and requires further study. Therefore, it is helpful to identify ferroptosis related genes (FRGs) involved in AMI and explore their expression patterns and molecular mechanisms.

**Methods:**

The AMI-related microarray datasets GSE66360 and GSE61144 were obtained using the Gene Expression Omnibus (GEO) online database. GO annotation, KEGG pathway enrichment analysis and Protein-protein interaction (PPI) analysis were performed for the common significant differential expression genes (CoDEGs) in these two datasets. The FRGs were obtained from the FerrDb V2 and the differentially expressed FRGs were used to identify potential biomarkers by receiver operating characteristic (ROC) analysis. The expression of these FRGs was verified using external dataset GSE60993 and GSE775. Finally, the expression of these FRGs was further verified in myocardial hypoxia model.

**Results:**

A total of 131 CoDEGs were identified and these genes were mainly enriched in the pathways of “inflammatory response,” “immune response,” “plasma membrane,” “receptor activity,” “protein homodimerization activity,” “calcium ion binding,” “Phagosome,” “Cytokine-cytokine receptor interaction,” and “Toll-like receptor signaling pathway.” The top 7 hub genes ITGAM, S100A12, S100A9, TLR2, TLR4, TLR8, and TREM1 were identified from the PPI network. 45 and 14 FRGs were identified in GSE66360 and GSE61144, respectively. FRGs ACSL1, ATG7, CAMKK2, GABARAPL1, KDM6B, LAMP2, PANX2, PGD, PTEN, SAT1, STAT3, TLR4, and ZFP36 were significantly differentially expressed in external dataset GSE60993 with AUC ≥ 0.7. Finally, ALOX5, CAMKK2, KDM6B, LAMP2, PTEN, PTGS2, and ULK1 were identified as biomarkers of AMI based on the time-gradient transcriptome dataset of AMI mice and the cellular hypoxia model.

**Conclusion:**

In this study, based on the existing datasets, we identified differentially expressed FRGs in blood samples from patients with AMI and further validated these FRGs in the mouse time-gradient transcriptome dataset of AMI and the cellular hypoxia model. This study explored the expression pattern and molecular mechanism of FRGs in AMI, providing a basis for the accurate diagnosis of AMI and the selection of new therapeutic targets.

## Introduction

Acute myocardial infarction (AMI), with its high morbidity and mortality, is one of the leading causes of death and disability among the middle-aged and elderly worldwide (1).

AMI is often characterized by destructive atherosclerotic plaques superimposed on acute thrombosis, and is the result of ischemic myocardial necrosis caused by acute interruption of myocardial blood flow (2). AMI is the result of interaction between genetic and environmental factors, and risk factors include obesity, hypertension, diabetes, and hypercholesterolemia (3). Myocardial cell death plays an important role in the development of AMI. Dead cardiomyocytes cannot be replaced by living cardiomyocytes because of their very limited potential for regeneration and repair (4). The intervention of myocardial cell death is important to improve the prognosis of AMI. Immediate restoration of coronary blood flow, that is, early successful reperfusion therapy, including thrombolysis or percutaneous coronary intervention (PCI), is the key measure to reduce myocardial injury and improve the prognosis of AMI patients (5). At present, ECG results and high-sensitive Cardiac troponin I are often used as the diagnosis basis of AMI, but they still have certain defects in the identification of early myocardial infarction, mild myocardial injury or stable coronary artery disease (6). Therefore, finding new biomarkers of AMI and studying the molecular mechanisms of myocardial cell death are of great clinical significance for the early diagnosis and treatment of AMI.

The term “ferroptosis” was first reported in 2012 and was considered to be an iron-dependent non-apoptotic cell death (7). Ferroptosis is a form of regulatory cell death (RCD) caused by iron-dependent lipid peroxidation and is different from other RCD patterns such as apoptosis, autophagy, pyroptosis and necroptosis (8). Ferroptosis is mainly manifested by decreased GPX4 and inhibition of “System Xc-” activity, which is influenced by various metabolic related pathways, including redox homeostasis, iron metabolism, sugar, lipid, and amino acid metabolism and mitochondrial activity, etc. (9–12).

Ferroptosis participates in the occurrence and development of a variety of human diseases, and its research in the field of cardiovascular disease is gradually deepening. Studies have shown that the formation and progression of atherosclerosis is associated with ferroptosis, and ferroptosis inhibitors can reduce the formation of atherosclerosis and reduce endothelial dysfunction (13, 14). One study showed that that ferroptosis was involved in doxorubicin induced cardiotoxicity and ischemia-reperfusion (I/R) mediated heart failure via the Nrf2/Hmox1 pathway (15), another study showed that ubiquitin-specific protease 7 (USP7) was involved in the regulation of ferroptosis by activating the p53/TfR1 pathway during myocardial I/R injury (16). These results suggest that the biological process of ferroptosis is deeply related to cardiovascular disease and ferroptosis-related genes may be new biomarkers or therapeutic targets for cardiovascular disease.

**FIGURE 1:**
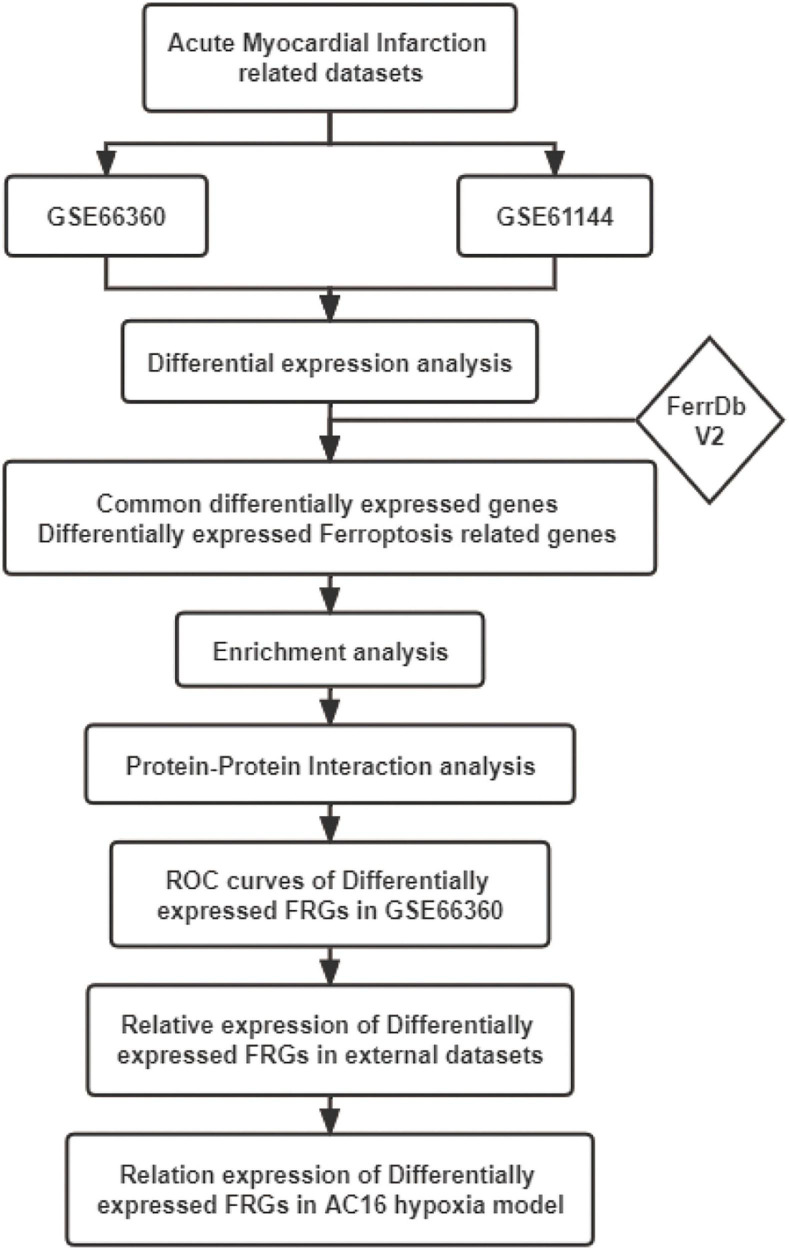
Flowchart of the steps performed in this study.

The mechanism of ferroptosis involved in the regulation of AMI is still not very clear. This study was performed to identify novel AMI related FRGs. AMI related datasets GSE66360, GSE61144, GSE60993, and GSE775 were downloaded and then analyzed using Bioinformatics analysis. The expression patterns of FRGs in AMI were further identified based on the original gene expression data in these datasets. ROC analysis was performed to identify the diagnostic value of these FRGs in AMI. These identified FRGs were further verified in the cell hypoxia model. Our study will explore the molecular mechanism of ferroptosis in AMI, so as to guide the individualized diagnosis and treatment of AMI. The workflow of the specific analysis is shown in Figure 1.

## Materials and methods

### Data resource

The Gene Expression Omnibus (GEO) database^1^ was used to download the AMI related microarray datasets GSE66360 (mRNA), GSE61144 (mRNA), GSE60993 (mRNA), and GSE775 (mRNA). GSE66360 comes from the GPL570 platform ([HG-U133_Plus_2] Affymetrix Human Genome U133 Plus 2.0 Array). GSE61144 comes from the GPL6106 platform (Sentrix Human-6 v2 Expression BeadChip). GSE60993 comes from the GPL6884 platform (Illumina HumanWG-6 v3.0 expression beadchip). GSE775 comes from the GPL81 platform ([MG_U74Av2] Affymetrix Murine Genome U74A Version 2 Array). The samples in GSE66360, GSE61144 and GSE60993 were all derived from human blood and grouped into healthy control and AMI groups. The samples in GSE775 were derived from myocardial tissue of myocardial infarction mice. Groups in GSE775 were divided according to different time of infarction (1, 4, 24, 48 h, 1, and 8 week), and each group contained three sham operation mice and three infarction mice. Table 1 shows the information of these 4 datasets obtained from the GEO database.

**TABLE 1:** The information of the 4 microarray datasets obtained from the GEO database.

Data source	Organism	Platform	Year	Sample source	Sample size (AMI/CON)	Detected RNA type
GSE66360	Homo Sapiens	GPL570	2015	Blood	49/50	mRNA
GSE61144	Homo Sapiens	GPL6106	2015	Blood	7/10	mRNA
GSE60993	Homo Sapiens	GPL6884	2015	Blood	7/7	mRNA
GSE775	Mus musculus	GPL81	2003	Tissue	18/18	mRNA

### Differential expression analysis

Raw sequencing data of GSE66360 and GSE61144 were downloaded from GEO database. GEO2R^2^ was used to perform the preliminary differential expression analysis. Benjamini-Hochberg method was used to correct adj. P for potential false positive results and adj. *P*-value < 0.05 and | logFC| > 0.8 was set as the cut-off criteria of DEGs. Venn diagram web tool^3^ was used to identify the CoDEGs in GSE66360 and GSE61144.

### Identification of differential expression genes associated with ferroptosis

FerrDb V2^4^ is the world’s first database that dedicates to ferroptosis regulators and ferroptosis-disease associations. A total of 431 regulatory factors including drivers, suppressors and markers were downloaded from FerrDB V2 database (Supplementary File 1). The DEGs in GSE66360 and GSE61144 were intersected with the genes obtained from FerrDB V2 to obtain DEGs associated with Ferroptosis. Based on the raw sequencing data in GSE66360 and GSE61144, the heatmap of these differentially expressed FRGs were produced using the gg plots package of R software (version: x64 3.2.1) (17).

### Functional and pathway enrichment analysis

The online biological tool, Database for Annotation, Visualization, and Integrated Discovery (DAVID; version6.8)^5^ was used to perform Gene Ontology (GO) analysis and Kyoto Encyclopedia of Genes and Genomes (KEGG) pathway enrichment analysis of CoDEGs in GSE66360 and GSE61144. Pathways with *P* < 0.05 were considered statistically significant.

### Construction of the protein-protein interaction network and identification of hub genes

In order to understand the mechanism of interaction between CoDEGs in AMI, a protein-protein interaction (PPI) network was constructed by string database (version10.0)^6^. This obtained PPI network was further visualized using Cytoscape software (version 3.7.1). The Cytohubba plug-in of Cytoscape software was used to screen out the top 10 key genes with high connectivity in this PPI network using the Maximal Clique Centrality (MCC) algorithm (18, 19). Multiple algorithms (Degree, EPC, Betweenness) continued to be used for prediction, and genes predicted by more than three methods at the same time are considered to be Hub Genes for AMI.

### Validation of the expression of ferroptosis marker GPX4 in multiple acute myocardial infarction related datasets

GPX4 is significantly downregulated during ferroptosis occurrence and is a well-recognized marker of the ferroptosis pathway. The expression of GPX4 was validated in multiple datasets (GSE60993, GSE61144, and GSE775) to confirm whether ferroptosis was associated with AMI or not.

### Further validation of differentially expressed ferroptosis related genes in external dataset GSE60993

GSE60993 was an AMI-related sequencing dataset comprising 7 healthy subjects, 9 patients with unstable angina (UA), 10 patients with non-ST segment elevation myocardial infarction (NSTEMI), and 7 patients with ST segment elevation myocardial infarction (STEMI). Based on the relative gene expression data in GSE60993, the abovementioned differentially expressed FRGs in GSE66360 and GSE61144 were further verified to study their expression differences in coronary artery disease (CAD).

### Receiver operating characteristic curve analysis

Differentially expressed FRGs in validation dataset GSE60993 were considered as potential biomarkers for AMI. Based on the expression data of these FRGs in GSE66360, Graphpad Prism (version:8.0) software was used to compare the control and AMI groups. Independent sample *T*-test was used to compare the two groups and *P* < 0.05 was considered statistically significant. ROC analysis was used to evaluate the diagnostic value of these FRGs for AMI. FRGs with the area under the curve (AUC) ≥ 7.0 and *P* < 0.05 were screened as potential biomarkers.

### Further validation of differentially expressed ferroptosis related genes in external dataset GSE775

The GSE775 dataset included myocardial tissue transcriptome data at 1, 4, 24, 48 h, 1 and 8 week after myocardial infarction in mice. Based on their raw expression data in GSE775, the expression of FRGs were further verified to study the change of expression of FRGs in myocardial infarction tissues over time. The comparison between Sham operation group and AMI group was performed by independent sample *T* test. *P* < 0.05 was considered statistically significant.

### Cardiomyocyte cell line culture and treatment

The human cardiomyocyte AC16 cell line purchased from BeNa Culture Collection (Beijing, China) was cultured in Dulbecco’s modified Eagle’s medium (DMEM, Gibco) with 10% fetal bovine serum (FBS, Gibco) and 1% penicillin-streptomycin (Sigma, St. Louis, MO, USA) at 37°C with 5% CO_2_. The three-gas incubator was used to establish an anoxic environment with 1% O_2_, 5% CO_2_, and 94% N_2_. The AC16 cardiomyocytes were exposed to 24 h of this condition to generate a hypoxia model. AC16 cells in the control group were under normoxia condition (21% O_2_, 5% CO_2_, and 74% N_2_) all the time.

### Real-time quantitative polymerase chain reaction

The expression of these FRGs in AC16 hypoxia cell model was further verified by qPCR. According to the manufacturer’s protocol, total RNA was isolated using FastPure^®^ Cell/Tissue Total RNA Isolation Kit V2 (Vazyme, Nanjing, China), and then synthesized with Hifair^®^ III 1st Strand cDNA Synthesis SuperMix for qPCR (Ye Sen, Shanghai, China). The Bio-Rad CFX96 Real-time PCR Detection System was performed to carry out the qRT-PCR, using Hieff UNICON^®^ Universal Blue qPCR SYBR Green Master Mix (Ye Sen, Shanghai, China). β-actin was used as the reference gene and the relative fold change was calculated by the method of 2^–ΔΔCt^. Details of all primers were presented in Supplementary File 2. Data were presented as mean values ± standard error of mean (SEM) from at least three independent experiments. Independent sample *T* test was used to compare the normoxia group and hypoxia group. FRGs with *P* < 0.05 was considered statistically significant.

## Results

### Identification of differential expression genes and differentially expressed FRGs in acute myocardial infarction

**FIGURE 2:**
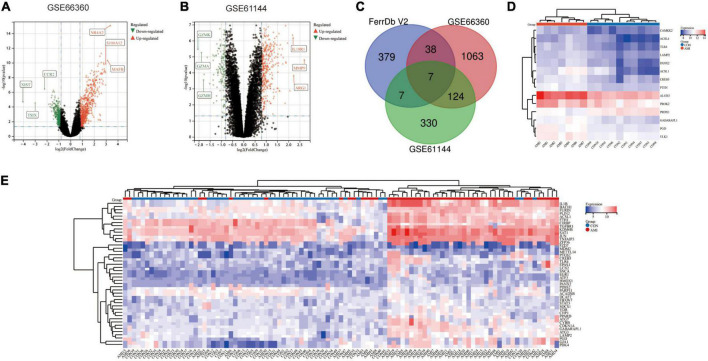
Identification of differentially expressed FRGs in GSE66360 and GSE61144. **(A)** The volcano plot of GSE66360. **(B)** The volcano plot of GSE61144. **(C)** Venn diagram of DEGs in GSE66360, GSE61144, and FRGs in FerrDb. **(D)** Heatmap of FRGs in GSE66360. **(E)** Heatmap of FRGs in GSE61144.

The AMI related datasets GSE66360 and GSE61144 were downloaded from the Gene Expression Omnibus (GEO) database. Based on the screening criteria mentioned above (*P* value < 0.05 and | logFC| > 0.8), DEGs in each dataset were screened out. There were 1232 DEGs, including 688 high-expressed, and 544 low-expressed, in GSE66360. There were 468 DEGs, including 325 high-expressed, and 143 low-expressed, in GSE61144. Supplementary File 3 presents detailed results of differential expression analysis. The top 5 upregulated DEGs and the top 5 downregulated DEGs in each dataset are shown in Table 2. The volcano plots of these DEGs in GSE66360 and GSE61144 are shown in Figures 2A,B. Based on the obtained DEGs and the ferroptosis related genes in FerrDB V2, the online venn tool was used to identify CoDEGs and differentially expressed FRGs (Figure 2C). A total of 131 CoDEGs were identified in GSE66360 and GSE61144. There are 45 and 14 differentially expressed FRGs in these two datasets respectively (Supplementary File 4). Seven ferroptosis related genes ACSL1, CREB5, GABARAPL1, LAMP2, PANX2, PGD, and TLR4 were significantly differentially expressed in both datasets. Figure 2D shows the heatmap of differentially expressed FRGs in GSE66360. Figure 2E shows the heatmap of differentially expressed FRGs in GSE61144.

**TABLE 2:** The top 5 upregulated DEGs and the top 5 downregulated DEGs in GSE66360 and GSE61144.

Dataset	Type	DEG	Expression	logFC	FDR
GSE66360	mRNA	NR4A2	up	3.37704444	7.83E-16
GSE66360	mRNA	MAFB	up	3.12991224	4.55E-11
GSE66360	mRNA	S100A12	up	2.98220317	1.26E-10
GSE66360	mRNA	CLEC7A	up	2.97162731	9.83E-08
GSE66360	mRNA	CXCL2	up	2.96654848	2.01E-09
GSE66360	mRNA	XIST	down	–4.0320135	2.33E-05
GSE66360	mRNA	TSIX	down	–2.9910711	3.35E-05
GSE66360	mRNA	CCR2	down	–2.1941029	4.15E-07
GSE66360	mRNA	LPAR5	down	–1.8493421	4.90E-05
GSE66360	mRNA	GIMAP8	down	–1.7295168	1.10E-04
GSE61144	mRNA	MMP9	up	2.70017256	1.55E-05
GSE61144	mRNA	ARG1	up	2.23048111	1.46E-04
GSE61144	mRNA	IL18R1	up	2.20219955	6.45E-07
GSE61144	mRNA	IL1R2	up	2.17550168	4.25E-07
GSE61144	mRNA	ORM1	up	2.05889349	1.30E-03
GSE61144	mRNA	GZMK	down	–2.0392509	3.56E-06
GSE61144	mRNA	GZMA	down	–1.8841985	5.76E-06
GSE61144	mRNA	GZMH	down	–1.7906003	2.93E-04
GSE61144	mRNA	NKG7	down	–1.7637924	1.32E-04
GSE61144	mRNA	EOMES	down	–1.6243454	1.14E-05

### Analysis of enrichment of common significant differential expression genes in GSE66360 and GSE61144

**FIGURE 3:**
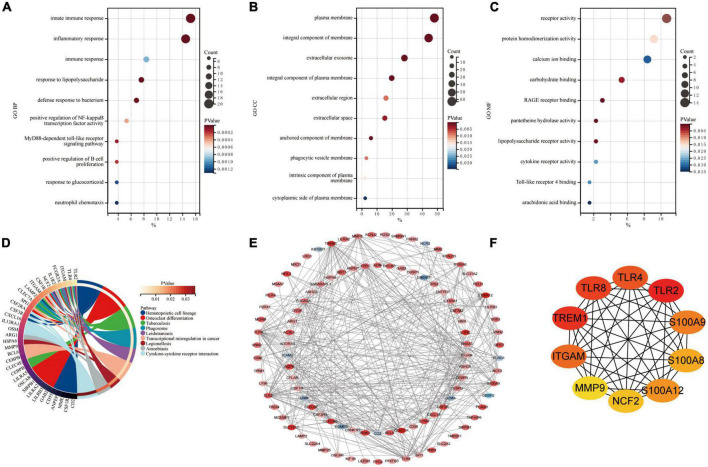
Enrichment analysis and PPI analysis of CoDEGs. **(A)** Bubble plot of biological process (BP; TOP10). **(B)** Bubble plot of cellular component (CC; TOP10). **(C)** Bubble plot of molecular function (MF; TOP10). **(D)** Circle plot of KEGG pathway enrichment analysis (TOP10). **(E)** The whole PPI Network. The 91 orange red nodes in the network represent up-regulated genes and the 10 blue nodes in the network represent down-regulated genes. **(F)** The top 10 genes predicted by the MCC algorithm.

Common DEGs in GSE66360 and GSE61144 were input into DAVID database for GO annotation and KEGG pathway enrichment analysis. Figures 3A–D shows the top 10 enriched GO annotation terms and KEGG pathways. For GO biological process (BP) analysis, the results showed that these CoDEGs are mainly enriched in the term of “innate immune response,” “inflammatory response,” “immune response,” “response to lipopolysaccharide,” and “defense response to bacterium” (Figure 3A). For GO cellular component (CC) analysis, the top 5 significantly enriched terms are “plasma membrane,” “integral component of membrane,” “extracellular exosome,” “integral component of plasma membrane,” and “extracellular region” (Figure 3B). For GO molecular function (MF) analysis, the top 5 significantly enriched terms are “receptor activity,” “protein homodimerization activity,” “calcium ion binding,” “carbohydrate binding,” “RAGE receptor binding” (Figure 3C). Furthermore, “Hematopoietic cell lineage,” “Phagosome,” “Cytokine-cytokine receptor interaction,” “Toll-like receptor signaling pathway,” and “TNF signaling pathway” are significant enrichment pathways in KEGG analysis (Figure 3D).

### Construction of the protein-protein interaction network and identification of hub genes in acute myocardial infarction

The CoDEGs were introduced into the String database. Based on the String database’s default execution criteria, a PPI network with 101 nodes and 370 edges was constructed and then visualized using Cytoscape software (Figure 3E). The 91 orange red nodes in this network represent high-expressed genes and the 10 blue nodes in this network represent low-expressed genes. The depth of the color represents the degree of upward or downward movement in the corresponding direction. Next, based on MCC algorithm, the top 10 genes (TLR2, TREM1, TLR8, TLR4, ITGAM, S100A9, S100A12, S100A8, NCF2, and MMP9) were screened out using the plug-in Cytohubba in Cytoscape (Figure 3F). KOBAS (version 3.0)^7^ online tool was used for further path enrichment analysis of these genes. The result shows that TLR2, TLR4, NCF2, and ITGAM are involved in “Phagosome” pathway, S100A8, S100A9, and MMP9 are involved in “IL-17 signaling pathway” pathway, TLR2, TLR4, and TLR8 are involved in “Toll-like receptor signaling pathway” pathway, NCF2, ITGAM, and MMP9 are involved in “Leukocyte transendothelial migration” pathway. In addition, the other three algorithms (Degree, EPC, Betweenness) were continued to be used for prediction, and eventually the genes predicted by at least three algorithms simultaneously were considered as Hub genes (Supplementary File 5 and Supplementary Figure 1). Finally, the top 7 hub genes ITGAM, S100A12, S100A9, TLR2, TLR4, TLR8, and TREM1 were considered as Hub Genes of AMI.

### Expression patterns of ferroptosis related genes in GSE66360 and GSE61144

**FIGURE 4:**
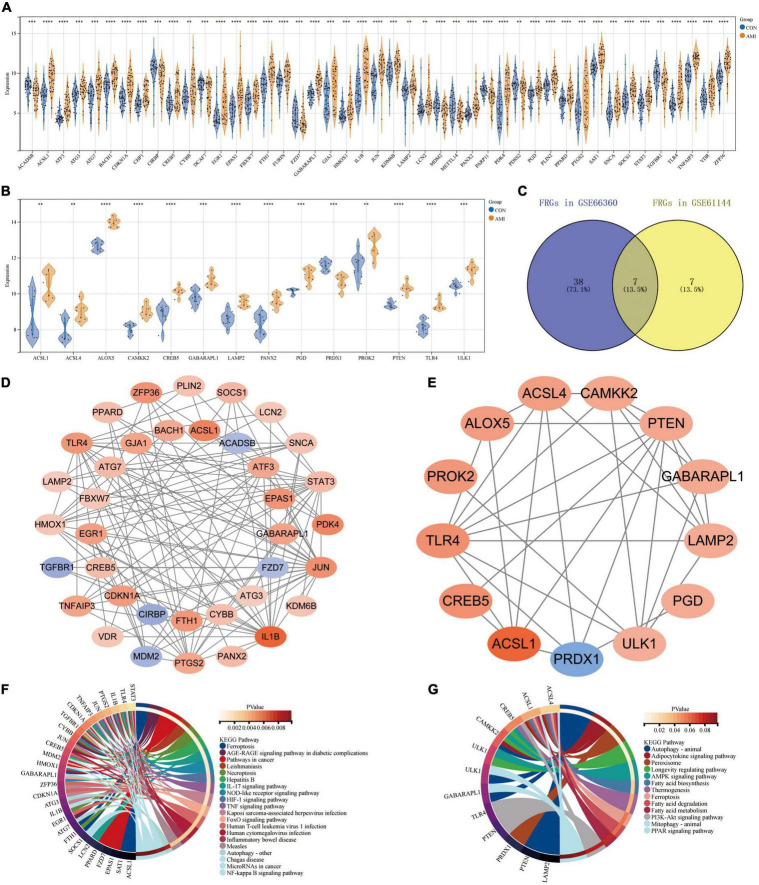
Expression patterns of FRGs in GSE66360 and GSE61144. **(A)** Expression patterns of FRGs in GSE66360. **(B)** Expression patterns of FRGs in GSE61144. **(C)** Venn diagram of FRGs in GSE66360 and GSE61144. **(D)** PPI network of FRGs in GSE66360. **(E)** PPI network of FRGs in GSE61144. **(F)** Circle plot of KEGG pathway enrichment analysis for FRGs on GSE66360. **(G)** Circle plot of KEGG pathway enrichment analysis for FRGs on GSE61144. **(A,B)** **P < 0.01; ***P < 0.001; ****P < 0.0001.

Based on their raw expression data in GSE66360 and GSE61144, FRGs in healthy and AMI patients were compared and analyzed. The results show that there are 45 and 14 FRGS in the dataset GSE66360 and GSE61144, respectively. The 45 FRGs in GSE66360 were all significantly differentially expressed, with high expression of all genes except for the low expression of nine genes, ACADSB, CIRBP, DCAF7, FZD7, MDM2, METTL14, PARP11, PDSS2, and TGFBR1 (Figure 4A). All 14 FRGs in GSE61144 were significantly increased except for the low expression of PRDX1 (Figure 4B). Figure 4C shows the Venn results of FRGs in these two datasets. Further PPI analysis and enrichment analysis were performed on FRGs mentionedabove. Figure 4D, consisting of 37 nodes and 121 edges, is the PPI network constructed by 45 FRGS identified in GSE66360. Similarly, Figure 4E, with 13 points and 33 edges, is the PPI network constructed by 14 FRGS identified in GSE61144. KEGG pathway enrichment analysis shows that FRGs in GSE66360 are significantly enriched in the term of “Ferroptosis,” “Necroptosis,” “NOD-like receptor signaling pathway,” “AGE-RAGE signaling pathway in diabetic complications,” and “Autophagy” (Figure 4F). FRGs in GSE61144 are significantly enriched in the term of “Autophagy,” “Adipocytokine signaling pathway,” “Peroxisome,” “AMPK signaling pathway,” “Fatty acid biosynthesis,” “Ferroptosis,” “Fatty acid metabolism,” “PI3K-Akt signaling pathway,” and “PPAR signaling pathway” (Figure 4G).

### Validation of the expression of ferroptosis marker GPX4 in multiple acute myocardial infarction related datasets

**FIGURE 5:**
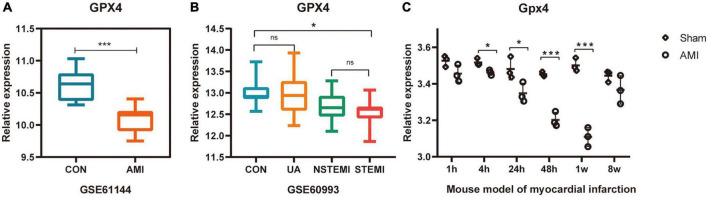
Validation of the expression of ferroptosis marker GPX4 in multiple AMI related datasets. **(A–C)** *P < 0.05; ***P < 0.001.

The expression of GPX4 was verified in datasets GSE61144, GSE60993, and GSE775. As predicted, the expression of GPX4 was significantly down-regulated in all three datasets (Figures 5A–C), suggesting that the biological process of ferroptosis may be involved in AMI.

### Further validation of differentially expressed ferroptosis related genes in external dataset GSE60993

**FIGURE 6:**
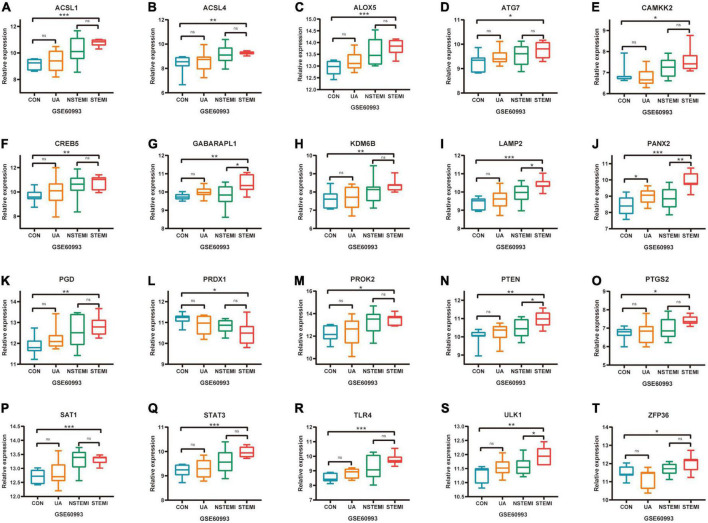
Further validation of differentially expressed FRGs in external dataset GSE60993. **(A–T)** *P < 0.05; **P < 0.01; ***P < 0.001.

The expression of 52 FRGs in GSE66360 and GSE61144 were further verified in external dataset GSE60993 (Figures 6A–T). Compared with healthy controls, a total of 19 FRGs, ACSL1, ACSL4, ALOX5, ATG7, CAMKK2, CREB5, GABARAPL1, KDM6B, LAMP2, PANX2, PGD, PROK2, PTEN, PTGS2, SAT1, STAT3, TLR4, ULK1, and ZFP36 were significantly up-regulated in STEMI patients, but only PRDX1 was down-regulated in STEMI patients. In addition, the expression of PANX2 was significantly different between CON and UA groups, the expression of GABARAPL1, LAMP2, PANX2, PTEN, and ULK1 was significantly different between NSTEMI and STEMI groups.

### Receiver operating characteristic curve analysis

**FIGURE 7:**
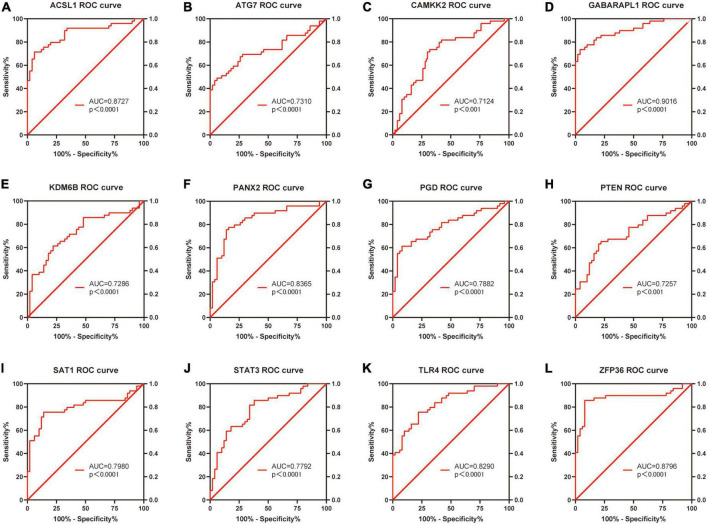
Receiver operating characteristic curve analysis of the top 12 ranked AUC values.

Based on the raw expression data in GSE66360, ROC analysis was performed on these 20 differentially expressed FRGs (ACSL1, ACSL4, ALOX5, ATG7, CAMKK2, CREB5, GABARAPL1, KDM6B, LAMP2, PANX2, PGD, PRDX1, PROK2, PTEN, PTGS2, SAT1, STAT3, TLR4, ULK1 and ZFP36) and AUC values were calculated. Finally, the AUC of these 20 FRGs were all greater than 0.6, and 13 of them, ACSL1 (AUC = 0.8727), ATG7 (AUC = 0.7310), CAMKK2 (AUC = 0.7124), GABARAPL1 (AUC = 0.9016), KDM6B (AUC = 0.7286), LAMP2 (AUC = 0.7045), PANX2 (AUC = 0.8365), PGD (AUC = 0.7882), PTEN (AUC = 0.7257), SAT1 (AUC = 0.7980), STAT3 (AUC = 0.7792), TLR4 (AUC = 0.8290), and ZFP36 (AUC = 0.8796), with AUC ≥ 0.7 were identified as potential markers of AMI. Figures 7A–L shows the ROC analysis results of the top 12 AUC values. The ROC analysis results for the remaining eight FRGs are shown in Supplementary File 5 and Supplementary Figure 2.

### Further validation of differentially expressed ferroptosis related genes in external dataset GSE775

**FIGURE 8:**
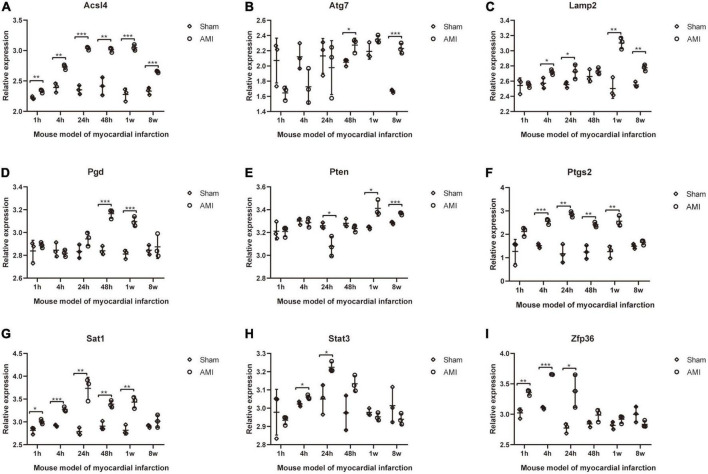
Further validation of differentially expressed FRGs in Mouse model of myocardial infarction dataset GSE775. **(A–I)** *P < 0.05; **P < 0.01; ***P < 0.001.

**FIGURE 9:**
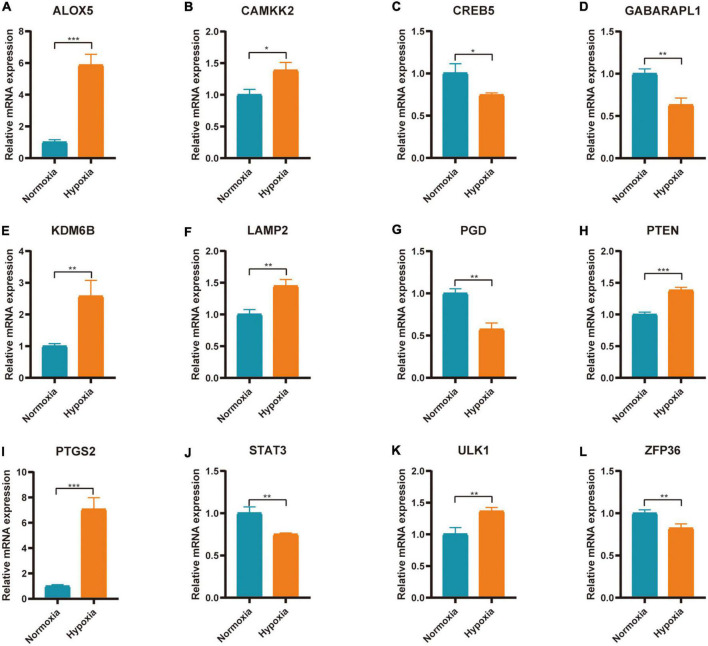
Expression of FRGs in AC16 cell hypoxia model. **(A–L)** *P < 0.05; **P < 0.01; ***P < 0.001.

The expression of 20 differentially expressed FRGs (ACSL1, ACSL4, ALOX5, ATG7, CAMKK2, CREB5, GABARAPL1, KDM6B, LAMP2, PANX2, PGD, PRDX1, PROK2, PTEN, PTGS2, SAT1, STAT3, TLR4, ULK1, and ZFP36) were further verified to study the expression changes of these FRGs in myocardial infarction tissues with time. As shown in Figures 8A–I, 9 FRGs (ACSL4, ATG7, LAMP2, PGD, PTEN, PTGS2, SAT1, STAT3, and ZFP36) were significantly differentially expressed at least twice at the six test time points (1, 4, 24, 48 h, 1, and 8 week after myocardial infarction). All these FRGs showed an upward trend in expression. The expression of STAT3 and ZFP36 was different only within 24 h after myocardial infarction. The differences in ATG7 expression and PGD expression did not occur until more than 48 h after myocardial infarction. The expression of ACSL4, LAMP2, PTEN, PTGS2, and SAT1 were different in the early and late stages of myocardial infarction.

### qRT-PCR verification of ferroptosis related genes in AC16 hypoxia model

The expression of 20 differentially expressed FRGs (ACSL1, ACSL4, ALOX5, ATG7, CAMKK2, CREB5, GABARAPL1, KDM6B, LAMP2, PANX2, PGD, PRDX1, PROK2, PTEN, PTGS2, SAT1, STAT3, TLR4, ULK1, and ZFP36) were further verified to study the effect of hypoxia on FRGs. The results showed 12 of them were differentially expressed (Figures 9A–L). They were ALOX5 (up), CAMKK2 (up), CREB5 (down), GABARAPL1 (down), KDM6B (up), LAMP2 (up), PGD (down), PTEN (up), PTGS2 (up), STAT3 (down), ULK1 (up), and ZFP36 (down). The expression of ALOX5, CAMKK2, KDM6B, LAMP2, PTEN, PTGS2, and ULK1 were consistent with the predicted result.

## Discussion

Acute myocardial infarction (AMI) is one of the main causes of sudden cardiac death in middle-aged and elderly people (20). Early reperfusion therapy is still a key measure for the treatment of AMI. Studies have shown that early restoration of blood perfusion can help reduce the size of myocardial infarction and reduce the incidence of arrhythmia and heart failure (21, 22). Therefore, it is of great importance to further study the pathogenesis of AMI and to find new biomarkers for early mild myocardial injury. Ferroptosis as a modality of regulatory cell death has received extensive attention in recent years. The understanding of the mechanism of ferroptosis in AMI is still limited. We used bioinformatics method to mine information from existing AMI related sequencing datasets, aiming to explore the expression patterns and molecular mechanisms of FRGs in AMI.

In the present study, 131 CoDEGs were screened in AMI-related datasets GSE66360 and GSE61144. These genes were mainly enriched in the pathways of “inflammatory response,” “immune response,” “plasma membrane,” “receptor activity,” “protein homodimerization activity,” “calcium ion binding,” “Phagosome,” “Cytokine-cytokine receptor interaction,” and “Toll-like receptor signaling pathway.” This suggests that inflammatory response is deeply involved in the pathogenesis of AMI. In the early stages of AMI, the injury is caused by a reduced blood supply to the tissue, and as cell death increases, the inflammatory response intensifies, which in turn further promotes cell death (23). In addition, it has also been shown that these pathways are associated with ferroptosis. The ferroptosis inhibitor ferrostatin-1 can inhibit TLR4 and inhibit LPS-induced cardiac dysfunction through TLR4/NF-κB signaling pathway (24). A PPI network of these CoDEGs was analyzed by String database. Based on this PPI network, 7 hub genes, ITGAM, S100A12, S100A9, TLR2, TLR4, TLR8, and TREM1 were identified. These genes are also enriched in inflammation-related pathways. It is reported that ITGAM is a key immune-related gene, which is significantly increased in blood samples of patients with AMI (25). S100A9 and S100A12 are up-regulated in the process of AMI and can induce macrophage/microglia inflammation (26, 27). Shuangxinfang (psycho-cardiology formula, PCF) could inhibit this process and improve the heart function of mice with myocardial infarction (26). TREM-1 is a receptor of immunoglobulin superfamily, which has been proved to induce and amplify inflammation in the progress of acute and chronic diseases (28). A study shows that the risk of death, recurrent myocardial infarction and stroke is increased in AMI patients with high expression of TREM-1 (29). A large number of studies have confirmed that the inflammatory response in AMI plays a key role in determining the size of myocardial infarction, and sustained proinflammatory response can lead to poor left ventricular remodeling after myocardial infarction (30, 31). The TLRs, include TLR1, TLR2, TLR4, TLR5, TLR6, and TLR11, act as sensors to damage associated molecular patterns (DAMPs) [such as heat shock proteins (HSPs), HMGB1, fibronectin-end domain A (FN-EDA)] (32). Inhibition of TLR2 or TLR4 can reduce myocardial infarction area and inhibit myocardial remodeling (33, 34).

Studies have shown that ferroptosis and inflammatory responses can interact. Cells undergoing ferroptosis are inherently more immunogenic because they release inflammatory cytokines and DAMPs, skewing the milieu to a proinflammatory state (35). Ferroptosis can also potently induce inflammation through the release of IL-33 and HMGB1 (36, 37). Some inflammatory cytokines (such as TNF, PGE2, IL-1β, IL-6, and IL-1) have been demonstrated to directly affect GPX4 levels. For example, TNF treatment of cells resulted in continuous downregulation of GPX4 and may further trigger ferroptosis (36). Therefore, Ferroptosis and inflammation may complement each other and participate in the regulation of AMI.

In addition, 45 and 14 FRGs were identified in GSE66360 and GSE61144, respectively. Enrichment analysis showed that these genes were involved in ferroptosis-related pathways. FRGs ACSL1, ATG7, CAMKK2, GABARAPL1, KDM6B, LAMP2, PANX2, PGD, PTEN, SAT1, STAT3, TLR4, and ZFP36 were significantly differentially expressed in external dataset GSE66360 with AUC ≥ 0.7, and may be potential biomarkers of AMI. These FRGs were further verified by external dataset GSE60993 (human, blood), GSE775 (mouse, tissue) and AC16 hypoxia model. The results showed that ACSL4, ATG7, LAMP2, PGD, PTEN, PTGS2, SAT1, STAT3, and ZFP36 were highly expressed in blood of patients with AMI and myocardial tissue of mice after AMI, among which LAMP2, PTEN, and PTGS2 were highly expressed in blood of patients with AMI, myocardial tissue of mice after AMI and myocardial cell hypoxia model. ALOX5, CAMKK2, KDM6B, and ULK1 were only significantly highly expressed in blood of patients with AMI and myocardial cell hypoxia model.

The regulation of these genes on ferroptosis has been studied. LAMP2 (Lysosome-associated membrane glycoprotein 2) plays an important role in chaperone-mediated autophagy. Deficiency of LAMP2 increases the risk of reactive oxygen species-induced ferroptosis in retinal pigment epithelial cells (38). One study has shown that the loss of phosphatase and tensin homolog deleted on chromosome 10 (PTEN) function confers ferroptosis resistance in cancer cells, and the inhibition of the PI3K-AKT-mTOR signaling axis sensitizes cancer cells to ferroptosis induction (39). This indicated that PTEN has the function of promoting ferroptosis. ALOX5 (5-Lipoxygenase) is an iron-containing and non-heme dioxygenase that catalyzes the peroxidation of polyunsaturated fatty acids such as arachidonic acid (40). Another study has shown that N-acetylcysteine (NAC) targets ALOX5 derived toxic lipids and can synergize with prostaglandin E _2_ to inhibit ferroptosis and improve outcomes following hemorrhagic stroke in mice (41). ULK1 (unc-51 like autophagy activating kinase 1) plays an important role in autophagy regulation. It is reported that ferritinophagy is involved in Bisphenol A-induced ferroptosis of renal tubular epithelial cells through the activation of the AMPK-mTOR-ULK1 pathway (42). In addition, CAMKK2, KDM6B, and PTGS2 have all been studied to be related to ferroptosis. These genes are activated in the biological process of ferroptosis and participate in the regulation of ferroptosis biological process (43, 44). Therefore, these identified FRGs (ALOX5, CAMKK2, KDM6B, LAMP2, PTEN, PTGS2, and ULK1) may be potential ferroptosis related biomarkers of AMI. It is important to note that the association of these genes with ferroptosis and AMI requires further clarification.

This study is based on the analysis of transcriptome data available in public databases and therefore has certain limitations. We need to collect blood samples in the population for testing to verify the diagnostic value of these biomarkers. It is also necessary to establish cell models and animal models to further study the mechanism of ferroptosis in AMI.

## Conclusion

We identified 131 differentially expressed genes in blood samples from patients with AMI. These genes are mainly associated with inflammatory reactions. Analysis of human blood sample sequencing datasets and mouse myocardial infarction sequencing datasets as well as cardiomyocyte hypoxia experiments indicated that FRGs ALOX5, CAMKK2, KDM6B, LAMP2, PTEN, PTGS2, and ULK1 may be potential biomarkers of AMI.

## Data availability statement

The datasets presented in this study can be found in online repositories. The names of the repository/repositories and accession number(s) can be found in the article/Supplementary material.

## Author contributions

JW, HC, and ZL performed the data analysis and drafted the manuscript. CL, YH, and TZ prepared the figures and contributed toward the study design. HZ and YL optimized the analysis protocol and completed the experimental part. XH and JC revised the figures, designed and supervised the study. All authors have read and approved the final manuscript.

## Footnotes

1
https://www.ncbi.nlm.nih.gov/

2
https://www.ncbi.nlm.nih.gov/geo/geo2r/

3
http://bioinformatics.psb.ugent.be/webtools/Venn/

4
http://www.zhounan.org/ferrdb/current/

5
https://david.ncifcrf.gov

6
http://string-db.org

7
http://kobas.cbi.pku.edu.cn/genelist/

## Supplementary material

Supplementary Table 1 Ferroptosis-related genes in FerrDB V2.

Supplementary Table 2 The primer sequence information of qPCR experiment.

Supplementary Table 3 The detailed results of differential expression analysis.

Supplementary Table 4 Ferroptosis-related genes in GSE66360 and GSE61144.

## Abbreviations

AMI: Acute myocardial infarction
AUC: Area under the curve
BP: Biological process
CAD: Coronary artery disease
CC: Cellular component
CoDEGs: Common significant differential expression genes
DAMPs: Damage associated molecular patterns
DEG: Differentially expressed gene
FRGs: Ferroptosis-related genes
GEO: Gene Expression Omnibus
GO: Gene Ontology
I/R: Ischemia-reperfusion
KEGG: Kyoto Encyclopedia of Genes and Genomes
MF: Molecular function
NSTEMI: non-ST segment elevation myocardial infarction
PCI: Percutaneous coronary intervention
PPI: Protein-Protein Interaction
RCD: Regulatory cell death
ROC: Receiver operating characteristic
STEMI ST: segment elevation myocardial infarction
UA: Unstable angina.
