# Supplementary material for: Expression pattern and diagnostic value of ferroptosis-related genes in acute myocardial infarction
Source: Front Cardiovasc Med. 2022 Nov 3;9:993592. doi: 10.3389/fcvm.2022.993592 (PMC9669064; doi:10.3389/fcvm.2022.993592)

**Supplementary Figure 1.** **Results of three algorithms (Degree, EPC, Betweenness) predicting Hub Genes**


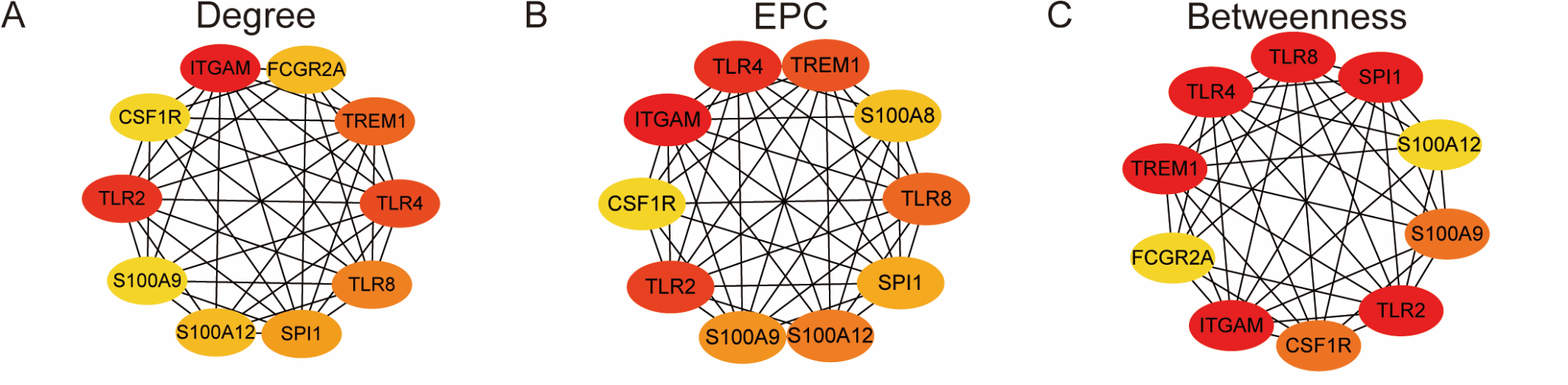


**Supplementary Figure 2. Receiver operating characteristic curve analysis of the bottom eight ranked AUC values**


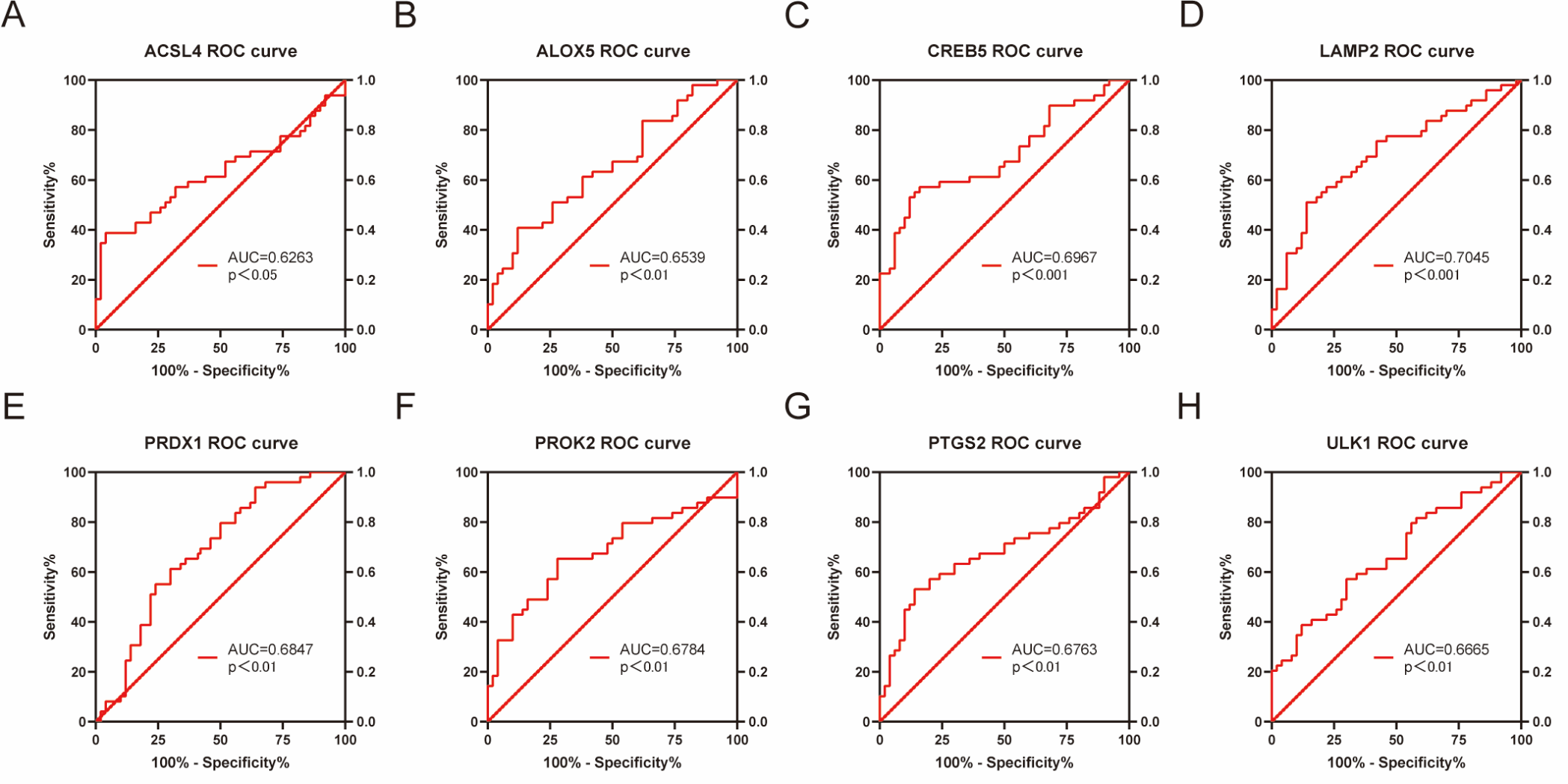

Supplement: Supplementary file 5 [file Data_Sheet_1.docx]
